# Surface-enhanced Raman spectroscopy introduced into the International Standard Organization (ISO) regulations as an alternative method for detection and identification of pathogens in the food industry

**DOI:** 10.1007/s00216-016-0090-z

**Published:** 2016-12-21

**Authors:** Evelin Witkowska, Dorota Korsak, Aneta Kowalska, Monika Księżopolska-Gocalska, Joanna Niedziółka-Jönsson, Ewa Roźniecka, Weronika Michałowicz, Paweł Albrycht, Marta Podrażka, Robert Hołyst, Jacek Waluk, Agnieszka Kamińska

**Affiliations:** 1 Institute of Physical Chemistry, Polish Academy of Sciences, Kasprzaka 44/52, 01-224 Warsaw, Poland; 2 Faculty of Biology, Institute of Microbiology, Applied Microbiology, University of Warsaw, Miecznikowa 1, 02-096 Warsaw, Poland; 3 Faculty of Mathematics and Natural Sciences, College of Science, Cardinal Stefan Wyszyński University, Dewajtis 5, 01-815 Warsaw, Poland

**Keywords:** *Salmonella* Typhimurium, SERS, ISO methods, Food, Bacteria detection, PCA

## Abstract

We show that surface-enhanced Raman spectroscopy (SERS) coupled with principal component analysis (PCA) can serve as a fast, reliable, and easy method for detection and identification of food-borne bacteria, namely *Salmonella* spp., *Listeria monocytogenes*, and *Cronobacter* spp., in different types of food matrices (salmon, eggs, powdered infant formula milk, mixed herbs, respectively). The main aim of this work was to introduce the SERS technique into three ISO (6579:2002; 11290–1:1996/A1:2004; 22964:2006) standard procedures required for detection of these bacteria in food. Our study demonstrates that the SERS technique is effective in distinguishing very closely related bacteria within a genus grown on solid and liquid media. The advantages of the proposed ISO-SERS method for bacteria identification include simplicity and reduced time of analysis, from almost 144 h required by standard methods to 48 h for the SERS-based approach. Additionally, PCA allows one to perform statistical classification of studied bacteria and to identify the spectrum of an unknown sample. Calculated first and second principal components (PC-1, PC-2) account for 96, 98, and 90% of total variance in the spectra and enable one to identify the *Salmonella* spp., *L. monocytogenes*, and *Cronobacter* spp., respectively. Moreover, the presented study demonstrates the excellent possibility for simultaneous detection of analyzed food-borne bacteria in one sample test (98% of PC-1 and PC-2) with a goal of splitting the data set into three separated clusters corresponding to the three studied bacteria species. The studies described in this paper suggest that SERS represents an alternative to standard microorganism diagnostic procedures.

**Electronic supplementary material:**

The online version of this article (doi:10.1007/s00216-016-0090-z) contains supplementary material, which is available to authorized users.

**Graphical Abstract:**
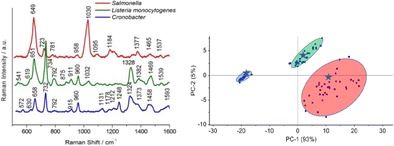
New approach of the SERS strategy for detection and identification of food-borne bacteria, namely *S. enterica*, *L. monocytogenes*, and *C. sakazakii* in selected food matrices

## Introduction

Many methods have been developed and applied in the detection and identification of bacteria species utilizing biochemical, immunological, and nucleic acid-based approaches [1]. However, these methods are time-consuming (at least 24 h to even 2 weeks), expensive because of the use of a variety of microbiological media, and require qualified personnel. Recently, real-time PCR assays for the detection of bacterial meningitis pathogens have been developed [2–4] and multiplex detection of several target DNAs is realizable [5]. Vibrational spectroscopy and fluorescence have also been employed for bacteria spore identification [6–8]. However, all these methods have some limitations, e.g., in the PCR technique the commonly used targets are unspecific and may cause false results, the fluorescence spectroscopic technique lacks specificity of the chemical information of analyzed samples, and IR spectroscopy is not suited for measurements in aqueous solutions. Therefore, there is an urgent need to develop a rapid, sensitive, simple, and reliable method for identification of pathogens.

The surface-enhanced Raman spectroscopy (SERS) is an optical method that can be used in testing of chemical and biochemical samples with high sensitivity and specificity. The enhanced signal is explained by the combination of electromagnetic (EM enhancement) and chemical (CT) mechanisms. The latter is related to charge transfer between a substrate and an adsorbed molecule [9]. The electromagnetic enhancement results from the resonance of the applied field with surface plasmon oscillations of the metallic nanostructures. Theoretically, the EM enhancement can reach factors of 10^3^–10^11^, whilst the CT enhancement factors have been calculated to be up to 10^3^ [10, 11]. This huge enhancement of Raman scattering (even single molecules can be observed [12]) ensures that SERS is very promising for biomedical and analytical studies. Moreover, this technique offers nondestructive, reliable, and fast detection, which leads to various practical applications in studying, for example, nucleic acids and proteins [13], therapeutic agents [14], drugs and trace materials [15], microorganisms [16], and cells [17]. Other important benefits of SERS include the quenching of the fluorescence background and improvement of the signal to noise ratio [18]. In particular, the development of SERS for the detection and identification of bacterial pathogens has attracted recent research efforts [19–25]. Rapid and early detection is potentially useful in clinical diagnosis, the food industry, or forensics.

In a pioneering study, Efrima and Bronk [26] presented the SERS spectra of *Escherichia coli* mixed with silver colloid and found that the recorded spectra are dominated by flavin vibrations. Flavins are important coenzymes present in the inner site of the bacteria cell wall [27]. The authors explained the specificity of SERS owing to enhanced binding affinities of silver nanoparticles to flavins via the isoalloxazine fused-ring moiety, which additionally works as a nucleation center for these nanoparticles. SERS spectra have also been reported for bacteria placed on electrochemically roughened metal surfaces [20, 21], bacteria coated by silver metal deposits [24, 25], or bacteria co-deposited with metal nanoparticles on a glass surface [28]. Spectral analysis allows one to study the bacteria structure, thus enabling the detection, diagnosis, and differentiation among bacteria species. Additionally, it may provide a considerable amount of detailed information, which is important for understanding the biological and chemical structure of the organisms.

An interesting issue is the detection of food-borne bacteria. *Salmonella enterica*, common bacteria found in rotten or unwashed food, is one of the most important food-borne pathogens worldwide and the second most frequently reported zoonotic agent in the European Union (EU) after thermotolerant *Campylobacter*. In 2014, a total of 88,238 confirmed salmonellosis cases were reported by 27 member states (MS) of the European Union, resulting in a notification rate of 23.4 cases per 100,000 population [29, 30]. Therefore the fast and simple detection of *Salmonella* in food is needed. Assaf et al. [31] demonstrated in 2014 the possibility of using normal Raman spectroscopy coupled with ISO standards for the detection of *Salmonella* spp. in selected food samples. However, principal component analysis (PCA) of Raman spectra reveals only 51% of total variance between bacteria species isolated from food industry samples. Another serious infection is listeriosis usually caused by eating food contaminated with *L. monocytogenes*. Listeriosis represents a serious public health problem since it has been fatal in around 20% of cases during the last two decades [32]. Infections caused by *Cronobacter sakazakii*, formerly *Enterobacter sakazakii*, are also dangerous, especially for older people and babies [33]. The Electronic Supplementary Material (ESM) presents the characteristics of these three bacteria species in more detail.

The identification methods of food-borne bacteria are standardized at the international level by the International Organization for Standardization (ISO) and mostly based on conventional microbiology. In this paper we show a new approach of using SERS technique instead of current identification process standards to detect food-borne bacteria, namely *Salmonella* spp., *Listeria monocytogenes*, and *C. sakazakii* (biochemical methods do not allow the identification of the species within the genus *Cronobacter*) in different types of food matrices (milk powder/infant formula, salmon, ham, eggs, mixed herbs) using Ag@FTO SERS substrates (Polish Patent Application P-408785).

We compare the SERS experiment with detection steps requested in ISO 6579:2002 [34] (horizontal method for the detection of *Salmonella* spp.), ISO 11290–1:1996/A1:2004 [35] (horizontal method for the detection and enumeration of *L. monocytogenes*), and ISO/TS 22964:2006 [36] (IDF/RM 210:2006) (a method for the detection of *E. sakazakii* in milk powder and powdered infant formula) standards. In the mentioned standards, the used methods allow one to detect one *Salmonella* spp. cell in 25 g of a food sample, one *L. monocytogenes* cell in 25 g of ready-to-eat foods intended for infants and for special medical purposes or in ready-to-eat foods able to support the growth of *L. monocytogenes*, and one *C. sakazakii* cell in 10 g of dried infant formula and dried dietary foods for special medical purposes intended for infants below 6 months of age. The mentioned ISO standards are adapted to Commission Regulation (EC) No. 2073/2005 of 15 November 2005 on microbiological criteria for foodstuffs.

The proposed SERS-based method of bacteria identification challenges the standard biochemical methods in terms of simplicity, specificity, and rapidity (the time of the whole analysis is reduced to 48 h from a total of about 140 h required by ISO standards). Additionally, the procedure presented in this study combines the SERS technique with multivariate statistical methods. To show the significant differences among SERS spectral features PCA as one of the most robust statistical methods is applied to (i) extract the biochemical information from bacteria spectra, (ii) perform the statistical classification of microorganisms, and finally (iii) identify the spectrum of an unknown sample by comparing it to the library of spectra from known bacteria.

## Materials and methods

### Bacteria strains and growth media

The following bacteria strains were used in this study: *L. monocytogenes* ATCC 13932, *L. ivanovii* PZH 7/04, *S. enterica* subspecies I, serovar Typhimurium 2021, *C. sakazakii* ATCC 29544. *L. ivanovii* PZH 7/04, and *Salmonella* Typhimurium 2021 were obtained from the collections of the National Institute of Public Health - National Institute of Hygiene (Warsaw, Poland).

Cultures were maintained in trypticase soy yeast extract agar (TSYEA) (Oxoid, Basingstoke, Hampshire, UK) at 4 °C throughout the study period and stored at −80 °C in brain heart infusion broth (BHI) supplemented with 20% glycerol. Half Fraser broth, Fraser broth, Chromogenic *Listeria* Lab-Agar acc. to ISO 11290 (Chrom Lis) and Palcam *Listeria* Lab-Agar (Palcam) were used to detect *L. monocytogenes*. Muller–Kauffmann tetrathionate novobiocine broth (MKTTn), Rappaport–Vassiliadis soya broth (RVS), xylose lysine deoxycholate agar (XLD), and Chromogenic *Salmonella* Lab-Agar (Chrom Sal) were used to detect *Salmonella* spp. Buffered peptone water (BPW), modified laurylsulfate-tryptose vancomycin broth (mLST), and ESIA Lab-Agar (ESIA) were used to detect *C. sakazakii* (formerly *E. sakazakii*). All media were purchased from Biocorp (Poland).

### Food samples

Five food matrices including smoked salmon (for detection of *L. monocytogenes* and *Salmonella* spp.), ham (for detection of *L. monocytogenes*), eggs (for detection of *Salmonella* spp.), powdered infant formula and mixed herbs (for detection of *Cronobacter* spp.) were analyzed. The food samples came from retail stores and were transported to the laboratory inside portable insulated cold boxes (except milk infant powder and spices). These transport conditions guarantee the chemical and biological stability of samples over time. The samples were immediately subjected to microbiological analysis.

For preparation of inoculum, colonies from 24-h cultures of *L. monocytogens* ATCC 13932, *S*. Typhimurium 2021, and *C. sakazakii* ATCC 29544 were resuspended in sterile saline solution to a turbidity of 0.5 McFarland units (approximate cell count density 1.5 × 10^8^ cfu) using densitometer (Densilameter II, Pliva-Lachema Diagnostika, Czech Republic). Bacterial suspensions were diluted in saline solution to 1 × 10^−6^ using a 10-fold serial dilution protocol.

Food samples of 25 g (smoked salmon, ham, and eggs) or 10 g (powdered infant formula and mixed herbs) were taken in an aseptic manner and homogenized in 225 ml and 90 ml, respectively, of pre-enrichment broth.

Each sample was performed in triplicate: (i) as a control, sample containing the food matrix and appropriate pre-enrichment medium. For positive samples, (ii) 0.1 ml of 1 × 10^−5^ and (iii) 1 × 10^−6^ dilutions of bacterial suspension were added to the pre-enrichment medium mixed with food samples.

The pre-enrichment, enrichment, and selective isolation steps were performed according to the standard procedures, ISO 11290–1 (*L. monocytogenes*), ISO 6579:2002 (*Salmonella* spp.), and ISO/TS 22964:2006 (*Cronobacter* spp.).

### ISO standards versus SERS-based methodology

**Fig. 1:**
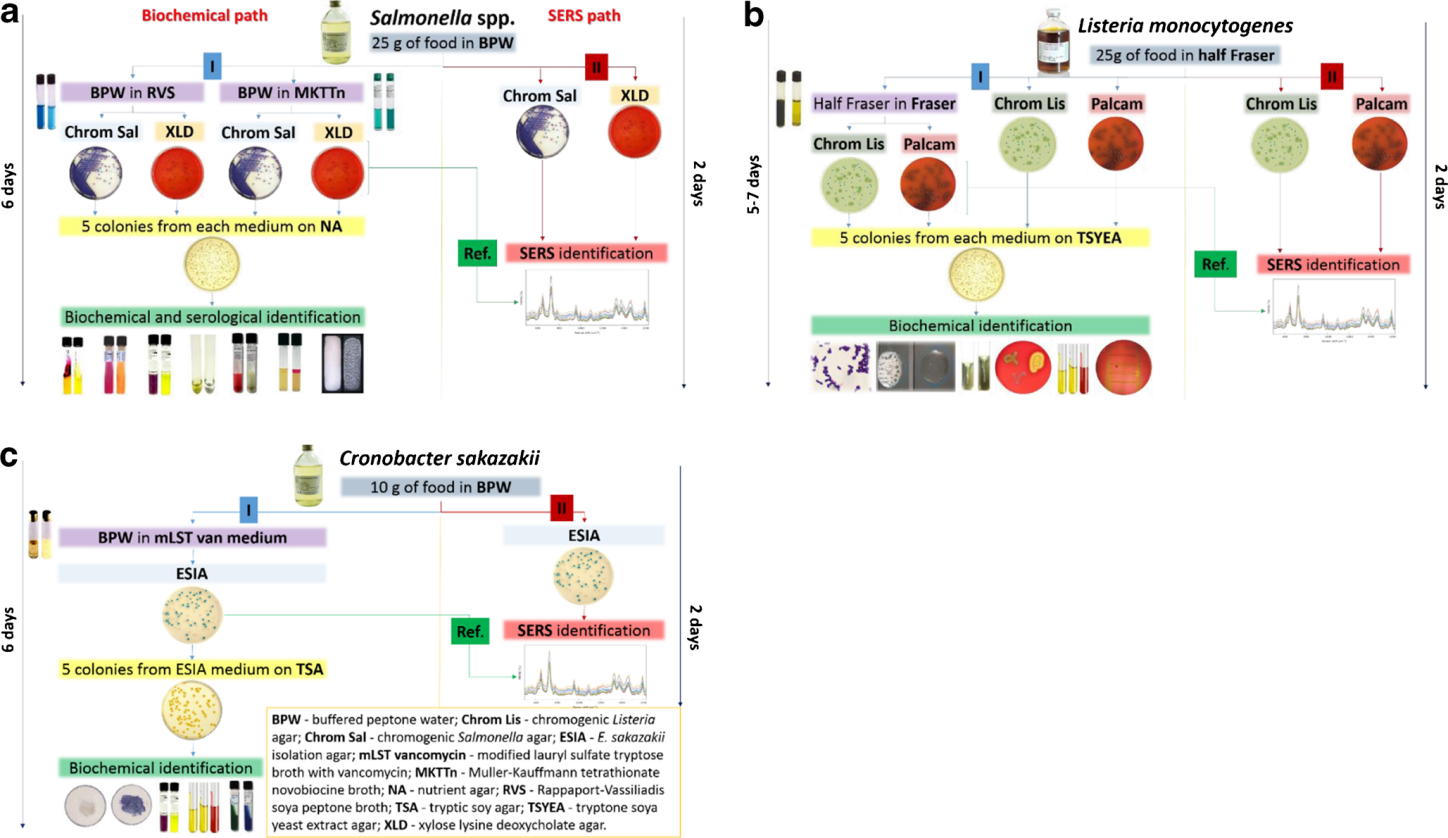
Scheme representing the different paths applied for **a** *Salmonella* spp., **b** *L. monocytogenes*, and **c** *C. sakazakii* detection in food samples

The SERS-based methodology for bacteria identification in respect to ISO standards is presented in Fig. 1 and Fig. S1 (see ESM).

#### *Salmonella* spp. detection

##### Path I (biochemical)

For *Salmonella* detection (ISO 6579:2002) the number of bacterial cells was increased in BPW mixed with 25 g of food sample. After that the simultaneous two enrichment steps in RVS (which is recommended as a selective enrichment medium for the isolation of *Salmonella* from food and environmental specimens) and MKTTn (which selectively promotes the growth of *Salmonella* and inhibits Gram-positive bacteria) were completed. These selective media reveal the presence of *Salmonella* colonies in food samples. Ten microliters of each selective medium is cultured on (1) XLD agar and on (2) Chrom Sal agar. Then, five suspect colonies from XLD and chromogenic *Salmonella* agar are incubated on nutrient agar (NA). After 15 h of incubation the colonies are identified within the next few days by biochemical methods to confirm or deny the presence of *Salmonella* in the analyzed sample (Fig. 1a).

##### Path II (ISO-SERS)

The shortest variant of *Salmonella* detection procedure from food samples provides the identification within only 48 h. Here 10 μl of the liquid part of BPW and food sample mixture was streaked directly onto the surface of XLD and Chrom Sal agar and was identified by the proposed SERS-based method (Fig. 1a).

##### Reference path (ISO-SERS)

After the pre-enrichment in BPW and selective enrichment in RVS and MKTTn, 10 μl of each selective media was rinsed and cultured on XLD agar and on Chrom Sal agar. This level ends the SERS-based method for *Salmonella* identification (Fig. 1a) and cuts the total time required by ISO to 72 h.

##### Reference from precultures

The bacterial stocks stored at −80 °C in brain heart infusion broth (BHI) supplemented with 20% glycerol were freshly cultured on appropriate media (*S*. Typhimurium on XLD and Chrom Sal media, *L. monocytogenes* and *L. ivanovii* on Chrom Lis and Palcam media, and *C. sakazakii* on ESIA).

Figure 1 presents in detail these three paths used for each bacteria identification. The procedures applied for *Salmonella* identification can be divided into several paths: (i) ISO recommended methods (144 h, path I), (ii) analysis of bacteria colonies from XLD and Chrom Sal media after pre-enrichment in BPW and selective enrichment in RVS and MKTTn media (72 h, Ref. path), and (iii) direct analysis of bacterial colonies from XLD and Chrom Sal media after pre-enrichment in BPW (48 h, path II). For all paths we grow Salmonella onto broths recommended by ISO standards.

#### *L. monocytogenes* detection

The procedure applied for *L. monocytogenes* identification can be divided into several similar paths as in the case of *Salmonella* spp.: (i) ISO recommended methods (120–168 h), (ii) direct analysis of bacterial colonies from Palcam and Chrom Lis media after selective enrichment in Half Fraser medium (48 h) and analysis of bacteria colonies from Palcam and Chrom Lis media after selective enrichment in Half Fraser and Fraser media (Ref. path 72 h) (Fig. 1b). For all paths we grow *L. monocytogenes* onto broths recommended by ISO standards. Half Fraser and Fraser media increase the number of *Listeria* spp. cells in samples. The whole procedure (according to path I) of *L. monocytogenes* detection and identification takes up to 7 days. Two other paths, i.e., path II and the Ref. path (novelty introduced in the SERS-based procedure), simplify the identification processes to 2 and 3 days, respectively.

In practice, for *L. monocytogenes* detection firstly the number of bacteria in Half Fraser broth mixed with 25 g of food sample was increased, and, the next day, 100 μl of this medium was transferred to Fraser broth and incubated in two variants: for 24 h and 48 h. Then, in each variant, bacteria were cultured on (i) Palcam with supplements (usually used as a selective and differential medium for the detection and isolation of *L. monocytogenes* from foods and environmental samples) and on (ii) Chrom Lis (medium for isolation, enumeration, and presumptive identification of *Listeria* species and *L. monocytogenes* from food samples). After 24 h the SERS spectra were collected for these two media.

*Listeria* is a genus of bacteria which encompasses several species, but only *L. monocytogenes* is regulated by Commission Regulation (EC) No. 2073/2005 [29] and should not be present in food samples. The detection system of Chrom Lis (ISO standard) is based on 5-bromo-4-chloro-3-indolyl-β-d-glucopyranoside, which can be cleaved by β-d-glucosidase produced by all *Listeria* spp. The second most typical pathogenic bacterium is *L. ivanovii*, but this is so far not listed in the aforementioned Commission Regulation. However, the information about bacteria species present in the sample is crucial. The two pathogenic species, *L. monocytogenes* and *L. ivanovii*, can be distinguished from non-pathogenic *Listeria* spp. by their phosphatidylinositol-specific phospholipase C (PI-PLC) activity [37]. The typical colony morphology of *Listeria* spp. is reported to be turquoise blue. Pathogenic *Listeriaceae* are additionally surrounded by a translucent halo [38].

#### *C. sakazakii* detection

Following the ISO/TS 22964:2006 (IDF/RM 210:2006) standard, five colonies from ESIA medium are cultured on tryptic soy agar (TSA) for 24 h and then identified by biochemical methods (Fig. 1c, path I, 144 h). In the proposed here SERS-based method (path II) 10 μl of the mixture of BPW and food sample was streaked directly onto the surface of ESIA plate and identified by SERS (Fig. 1c, path II). This path cuts the total time of the experiment to 48 h. In practice, for *C. sakazakii* identification, these bacteria were multiplied in the mixture prepared by dissolving 10 g of milk powder in BPW. The next day 100 μl of the obtained liquid was cultured in mLST medium. After 1 day of culturing, by using a 10-μl loop, the mixture was streaked onto the surface of the ESIA agar and incubated for one more day. This step ends the reference path of *C. sakazakii* identification (Fig. 1c, Ref. path) at 78 h.

### Chemometric analysis

PCA was performed on the preprocessed SERS spectra. PCA is a data reduction technique in which the new variables, called principal components (PC), are calculated from original variables. The first principal component (PC-1) accounts for the greatest variance in the data. The method of PCA is based on a model assuming *X* = *TP* ^T^ + *E*, where the *X* matrix is decomposed by PCA into two smaller matrices, one of scores (*T*) and another of loadings (*P*) [39], and *E* is the error matrix. PC scores are related to a linear combination of the original variables and describe the differences or similarities in the samples. PCA provides insight into the percentage of variance explained by each PC and shows how many PCs should be kept to maintain the maximum information from the original data without adding noise to the current information. Loadings describe the data structure in terms of variable correlation and reflect how well one PC takes into account the variation of that variable. By analyzing the plot of PC loadings as a function of the variables (i.e., Raman shifts) one can indicate the most important diagnostic variables or regions related to the differences found in the data set. In this study we applied PCA to all collected spectra of bacteria, namely *S. enterica*, *L. monocytogenes*, and *C. sakazakii*. This analysis enables one to investigate the spectral variations and to find the most significant modes contributing to the variance explained by these PCs. PCA was performed on the preprocessed Raman spectra to (a) evaluate the spectral differences among the bacteria species grown on XLD agar and on Chrom Sal agar (*Salmonella* spp.), (b) identify *Listeria* species (*L. monocytogenes* and *L. ivanovii*), and (c) identify *C. sakazakii* from among bacteria species grown on ESIA agar, and finally to (d) develop a model for detection of food-borne bacteria, namely *Salmonella* spp., *L. monocytogenes*, and *Cronobacter *spp.

## Experimental

### Bacteria sample preparation for SERS measurements

Single typical *S*. Typhimurium colonies on XLD and Chrom Sal agar, *C. sakazakii* on ESIA, and *L. monocytogenes* and *L. ivanovii* on Chrom Lis agar were collected and the bacteria were resuspended in a sterile saline solution and centrifuged for 5 min at 1200 × *g* in order not to destroy the cell membrane. Finally, the supernatant liquid was discarded and the bacterial cells were redispersed in 0.9% NaCl water. The centrifugation process was repeated three times to obtain a solution of clean bacterial cells. About 10 μl of aqueous bacterial solution was applied to the SERS substrate.

### Chemicals

Silver nitrate (AgNO_3_) and trisodium citrate dihydrate were purchased from Sigma–Aldrich; acetone, isopropanol, and methanol were purchased from Avantor Performance Materials Poland (POCH S.A., Poland). FTO-coated glass was from Delta Technologies. Water was purified with an ELIX system (Millipore, Merck, Germany). All reagents were used as received without further purification.

### Instrumentation

#### Preparation of SERS platform

SERS substrates were produced using a three-electrode electrochemical process with constant potential of −1.0 V applied for 15 min. Silver nanoparticles (AgNPs) were deposited on an FTO electrode from aqueous solution of 0.3 mM AgNO_3_ and 2.6 mM trisodium citrate dihydrate under controlled conditions of temperature and stirring. After electrodeposition, the electrodes with AgNPs were rinsed with deionized water and dried under a stream of air. The SERS spectra were collected from 40 different points for each sample in mapping mode (20 × 40 μm).

#### Raman spectroscopy and SERS

Measurements were carried out using a Renishaw inVia Raman system equipped with a 785-nm diode laser. The light from the laser passed a line filter and was focused on a sample mounted on an X–Y–Z translation stage with a ×50 microscope objective, NA = 0.25. The beam diameter was approximately 2.5 μm. The laser power at the sample was 5 mW or less. The microscope was equipped with 1200 grooves per mm grating, cutoff optical filters, and a 1024 × 256 pixel Peltier-cooled RenCam CCD detector, which allowed registering the Stokes part of Raman spectra with 5–6 cm^−1^ spectral resolution and 2 cm^−1^ wavenumber accuracy. The experiments were performed at ambient conditions using a back-scattering geometry.

The recording of the spectra was started immediately after placing the analyzed sample onto a SERS-active surface. During a period of about 30 min, SERS spectra were repeatedly recorded, while at the same time, the focus of the laser beam was readjusted. The time required for completing a single SERS spectrum was about 60 s. The obtained spectra were processed with the Wire3 software provided by Renishaw.

#### PCA spectral data analysis

SERS spectra were prepared for PCA using a two-step approach. First, using built-in OPUS software (Bruker Optic GmbH 2012 version) the spectra were smoothed with a Savitzky–Golay filter, the background was removed using baseline correction (concave rubberband correction; no. of iterations 10, no. of baseline points 64), and then the spectra were normalized using a Min–Max normalization. All the data were transferred to the Unscrambler software (CAMO software AS, version 10.3, Norway), where PCA was performed.

## Results

In this study the SERS technique was introduced into the ISO standards for identification of pathogenic bacteria in food, namely *Salmonella* spp., *L. monocytogenes*, and *Cronobacter* spp., in respect to the methodology presented in Fig. 1. According to Commission Regulation (EC) No. 2073/2005 *Salmonella* spp. should not be present in food samples in any amount, and *L. monocytogenes* or *Cronobacter* spp. should not be detected in selected food products. The identification procedures requested by ISO norms are complex and time-consuming (up to 6 days, see path I in Fig. 1a–c). As mentioned above, SERS has been used for fast identification of pathogens in the selected food samples (according to path II in Fig. 1a–c). In this analysis the long, time-consuming incubation is omitted. The direct SERS analysis (48 h) of bacteria colonies inoculated on agar with selective media (characteristic for incubated bacteria, see Fig. 1) was performed.

The longer path (72 h), named the Ref. path in Fig. 1, was applied to identify these three bacteria in respect to ISO standards and to validate the results obtained in path II. In the reference path the selective media along with selective enrichment allow one to grow only the colonies of the analyzed bacteria. The results from this step were used as a proof of identification made in path II. The data obtained in both the reference path and path II were additionally compared with the reference SERS spectra of all analyzed food-borne bacteria (*S*. Typhimurium, *L. monocytogenes*, *L. ivanovii*, *C. sakazakii*) collected from precultures (data not shown). All the obtained spectra (from reference path, path II, and precultures) allow identification of bacteria species (positive control) in the analyzed food samples using the SERS technique (ESM Fig. S4).

### SERS spectra

**Fig. 2:**
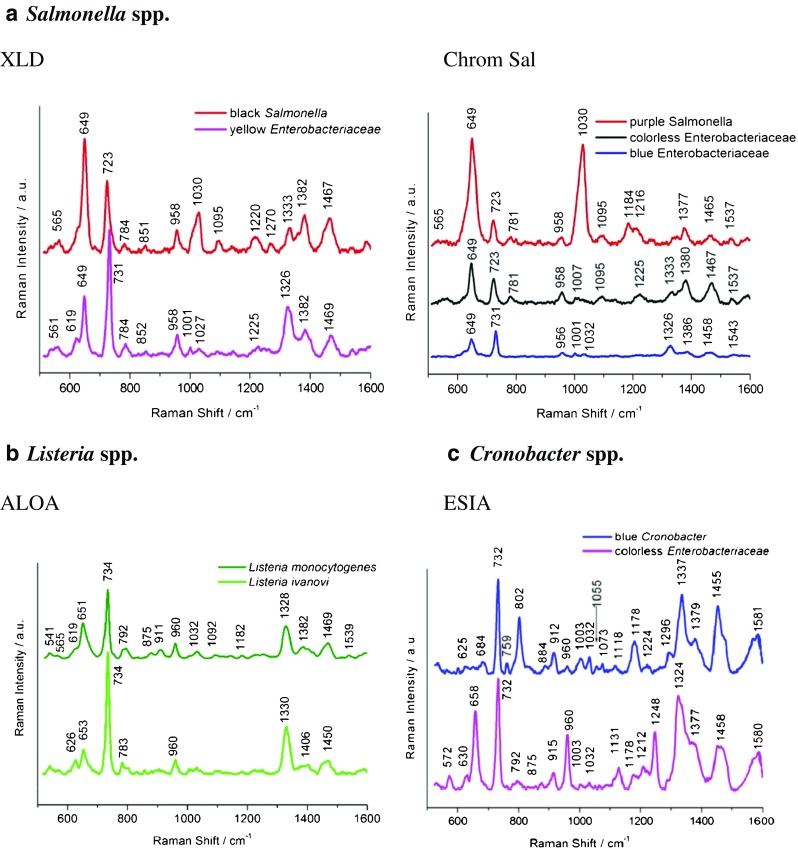
SERS spectra of *S*. Typhimurium cells and other bacteria species grown on XLD and chromogenic agars (**a**), *L. monocytogenes* and *L. ivanovii* detected in milk powder (infant formula), salmon, ham, and eggs (**b**), and *C. sakazakii* growing with *Enterobacteriaceae* (**c**) according to path II, see Fig. 1

According to Fig. 1a, the biochemical path (path I) of *Salmonella* spp. detection and identification takes 6 days. The novelty introduced in the ISO procedure by adding SERS (path II) reduces the identification process to 2 days. In path II, after culturing food samples (from salmon and eggs) contaminated with *Salmonella* cells on XLD and Chrom Sal agar, not only *Salmonella* colonies but also colonies of other bacteria species were obtained. On XLD the *Salmonella* colonies have a characteristic black color, while interfering *Enterobacteriaceae* strains are yellow. In the case of Chrom Sal agar *Salmonella* may also grow with two other interfering *Enterobacteriaceae* species which are colorless and blue, while *Salmonella* colonies are purple. SERS analyses of black or purple colonies of *Salmonella* and co-existing species were performed using Ag@FTO SERS-active substrates. Figure 2a presents the SERS spectra collected from *Salmonella* and other bacteria species grown on both these broths.

For *Salmonella* grown on both XLD and Chrom Sal media, several characteristic bands at 649, 723, 958, 1030, 1095, ca. 1220, and ca. 1467 cm^−1^ are observed. These bands are detected also in SERS spectra of many Gram-positive and Gram-negative bacteria species like *E. coli* or *S. epidermidis* [40, 41] and are assigned as follows: 649 cm^−1^ (guanine and tyrosine); 723 cm^−1^ (C–N stretching mode of the adenine part of flavin adenine dinucleotide, FAD); 958 cm^−1^ (C=C deformation or C–N stretching); 1030 cm^−1^ (C–C stretching); 1095 cm^−1^ (O–P–O stretching in DNA); 1220 cm^−1^ (amide III); 1467 cm^−1^ (CH_2_ deformation) [42]. The differences between these two spectra, especially in the ratio of intensities of some bands, e.g., 649, 723, and 1030 cm^−1^, originate from bacteria responding to environmental changes (XLD or Chrom Sal) by changing their metabolic profiles and composition of the cell walls [43].

Most of these bands appear also in the SERS spectra of bacteria co-existing with *Salmonella* (labeled yellow and blue *Enterobacteriaceae* in Fig. 2a). All bacteria species reveal their own individual spectral characteristics, which aids in the whole organism fingerprint analysis. For example, the band at 1030 cm^−1^ can be seen in *Salmonella*, but not in interfering *Enterobacteriacae* species. To distinguish *Salmonella* from these co-existing bacteria, the ratio of intensities of the bands at ca. 650 cm^−1^ and ca. 730 cm^−1^ can be used. Table 1 contains the assignments of all observed bands for *Salmonella*, *L. monocytogenes*, and *C. sakazakii* bacteria.

**Table 1:**
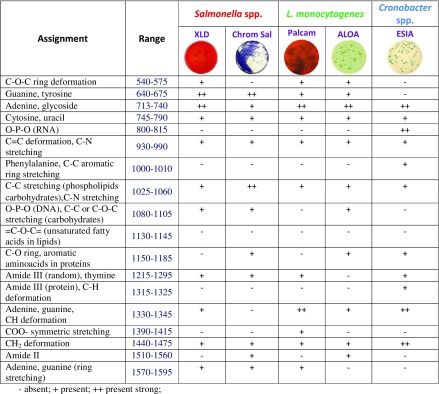
Main bands observed in *Salmonella *spp., *L. monocytogenes*, and *Cronobacter* spp. spectra and their assignments [28, 44–47]

- absent; + present; ++ strongly present

*L. monocytogenes* and *L. ivanovii* spectra (both species grown on ALOA agar from salmon and ham) presented in Fig. 2b are very similar; however, one can observe two main differences. In the case of *L. ivanovii* one can see an additional band at 626 cm^−1^ which is absent in the case of *L. monocytogenes*. Moreover, the intensity ratio of bands 734 and 650 cm^−1^ is higher in the *L. ivanovii* spectrum. Both spectra show also a common band at about 790 cm^−1^ (cytosine, uracil), 960 cm^−1^ (C=C deformation), and 1330 cm^−1^ (adenine, guanine, CH deformation). The detailed assignment of all the observed bands is presented in Table 1.

Subsequently culturing food samples contaminated with *C. sakazakii* cells on ESIA from powdered infant formula and mixed herbs, we obtained not only blue colonies of *C. sakazakii* but also white colonies of other bacterium species from the *Enterobacteriaceae* family (in case of mixed herbs). Figure 2c presents the differences between the SERS spectrum of *Cronobacter* cells and that other bacteria species grown on ESIA medium. One can notice, in both spectra, the presence of bands, characteristic for all bacteria species, at 732, 960, 1003, 1032, 1377, and 1455 cm^−1^ (CH_2_ deformation), but also additional bands present only in the case of *C. sakazakii*: 802 cm^−1^ (O–P–O in RNA) and 1337 cm^−1^.

The reproducibility of the recorded bacterial SERS signals is a crucial parameter for analytical and biomedical applications of this technique. Figure S7 (see ESM) shows an example of *C. sakazakii* SERS spectra recorded from different spots within the same sample. To obtain statistically valid results, the strong signal at 732 cm^−1^ was chosen to calculate the average standard deviation (AvSTD) and equals 15% based on the 30 SERS spectra recorded for the same platform. The average standard deviation of the SERS signals of *S. *Typhimurium and *L. monocytogenes* has also been calculated and is presented in the Table S1 (see ESM).

The SERS data were hereafter analyzed by chemometric methods to improve the accuracy of discrimination between these two very similar spectra.

### PCA

#### *Salmonella* spp.

**Fig. 3:**
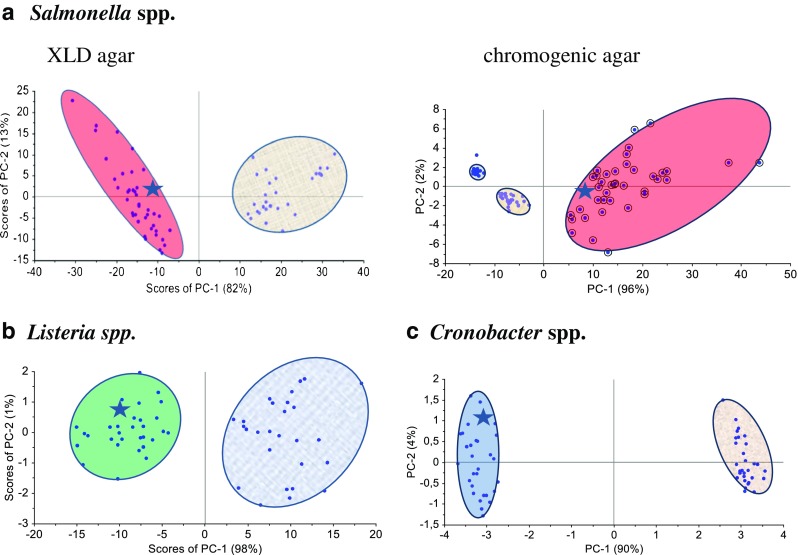
Scoreplots of PC-1 versus PC-2 component for **a** *Salmonella* Typhimurium (*red circle*), **b** *L. monocytogenes* (*green circle*) and *L. ivanovii* (*blue circle*), and **c** *C. sakazakii* (*navy blue circle*).* Asterisks* represent the scores calculated for test samples (smoked salmon—*Salmonella* spp., ham—*L. monocytogenes*, powdered infant formula—*Cronobacter* spp.)

PCA is used to build a model for classification of the closely related bacteria species. Initially, the analysis was performed over the whole spectral region between 500 and 1650 cm^−1^. In the first step we found that two principal components (PC-1, PC-2) are the most diagnostically significant and explain 84% and 95% of the variance in the data, for bacteria grown on XLD and Chrom Sal agar, respectively (Fig. 3a). The loadings of the PCs provide information on the variables (wavenumber of the spectrum) that are important for group separation. Figure S2 in the ESM displays the loadings plot of PC-1 for the whole wavenumber region. By analyzing these plots one can indicate the most important diagnostic variables in the analyzed data set. Variables with high loading values are the most important for diagnostic purposes. Moreover, the calculation of PCA in the area of the most pronounced marker bands at 649 cm^−1^ was performed. The PC-1 scores calculated for the region of the chosen marker give values of 82% and 96% of total variance and together with calculated PC-2 give 95% and 98% of total variance in respect to the studied samples (Fig. 3a and Table 2). These percentage values clearly discriminate the *Salmonella* species from other bacteria that grow independently in the same medium (XLD or Chrom Sal agar).

**Table 2:** Values of PCA scores calculated for analyzed bacteria species

Species	Range	Scores (%)	
		PC-1	PC-2
*S*. Typhimurium	XLD	82	13
	Chrom Sal	96	2
*L. monocytogenes*	ALOA	98	1
*Cronobacter* spp.	ESIA	90	4

#### *Listeria* spp.

PCA performed for *L. monocytogenes* and *L. ivanovii* (in the region of 500–1650 cm^−1^) gives the value of PC-1 equal to 83% of total variance (ESM Fig. S3b). In the next step the PCA calculation was performed in the chosen region, in the area of the most pronounced marker band at 734 cm^−1^ (ESM Fig. S3c). The PC-1 scores calculated for this region gives the value of 98% of total variance (Fig. 3b, ESM Fig. S3c, and Table 2). This result shows that PCA enables one to identify *L. monocytogenes* and *L. ivanovii* species with very high probability.

#### *Cronobacter* spp.

As in the case of *Salmonella* and *Listeria* species, PCA was performed for all the collected SERS data in the whole region (Fig. 2c) and in the areas of the most pronounced marker bands (ESM Fig. S4). The obtained PC-1 and PC-2 values yield 94% of total variance for *Cronobacter* spp. and *Enterobacteriacae* (Fig. 3c and Table 2). These percentage values clearly discriminate the *Cronobacter* species from other bacteria that grow independently on the same ESIA medium.

**Fig. 4:**
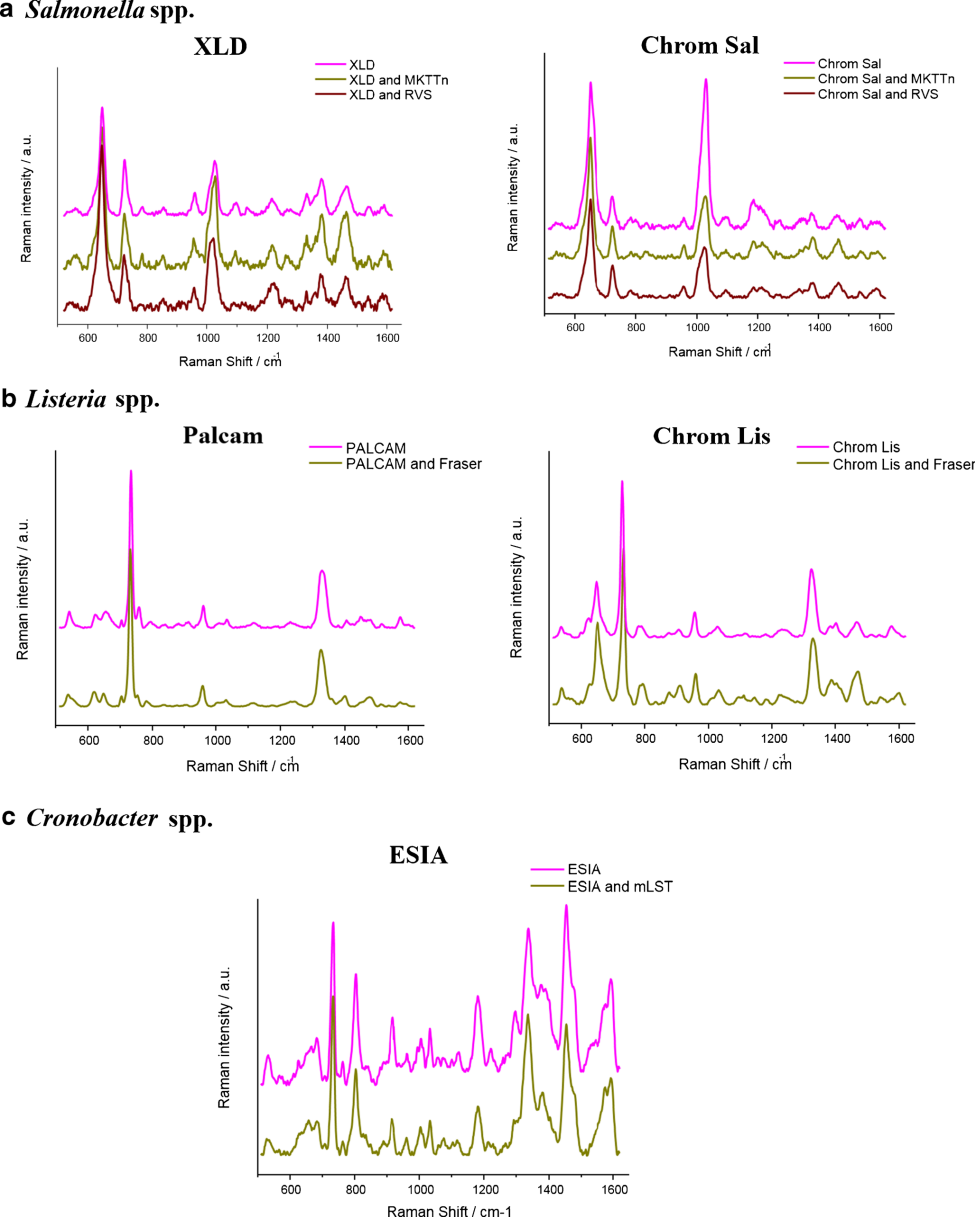
SERS spectra of **a** *Salmonella* spp., **b** *Listeria* spp., and **c** *Cronobacter* spp. obtained from path II, reference paths, and reference precultures

To validate the SERS discrimination among the tested bacteria, an additional step based on the reference SERS spectra was applied. Figure 4 displays the comparison among the SERS spectra of the analyzed bacteria obtained from path II, reference path, and reference precultures.

These results show no differences among the SERS spectra of particular bacteria species and confirm the significance of the proposed, simplified to 48 h, ISO-SERS method (path II). Additionally, using multivariate analysis we demonstrate the impact of SERS technique introduced into ISO standards. PCA was performed on a data set containing all references and path II SERS spectra of analyzed bacteria. PC scores obtained for reference SERS spectra are marked by asterisks in Fig. 3. As can be seen, the positions of these asterisks are in the area of PC clusters of *Salmonella*, *Listeria*, and *Cronobacter* species from path II. This demonstrates the ability to use SERS in identification and discrimination of these food-borne bacteria in the food industry.

**Fig. 5:**
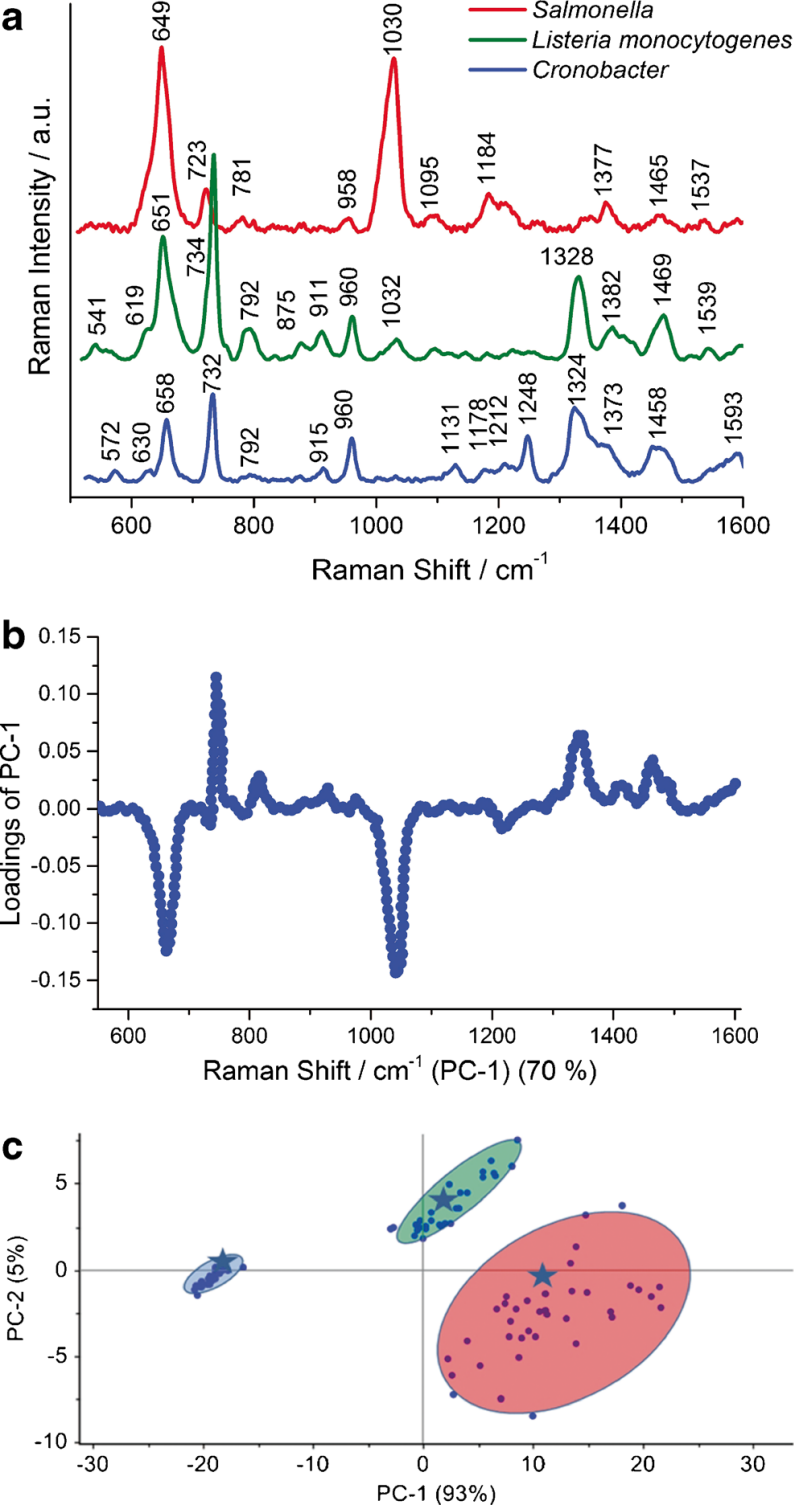
SERS spectra of *Salmonella* Typhimurium, *L. monocytogenes*, and *C. sakazakii* (**a**). Loadings plot of the first principal component showing the most prominent marker bands and **b** plot of the PC-1 versus PC-2 for the selected marker band at 650 cm^−1^ (**c**).* Asterisks* represent the scores calculated for test samples (smoked salmon—*Salmonella* spp., ham—*L. monocytogenes*, and powdered infant formula—*Cronobacter* spp.)

To check the utility of the ISO-SERS-based method for simultaneous detection and identification of three food-borne bacteria—*S. enterica*, *L. monocytogenes*, and *C. sakazakii* — in one sample test, PCA was performed. Figure 5a shows the spectral comparison of all three food-borne bacteria which are the subject of this study. These SERS spectra exhibit the same common spectral features for the majority of bacteria species, but with some differences in the band positions, relative intensity ratios, and/or appearance of new bands. These differences allow one to identify the particular bacteria species in different food samples.

The loading plot of PC-1 in relation to variables (Raman shift) calculated for these three bacteria indicates the most pronounced marker bands (650, 725, 1030 cm^−1^) which may be used in differentiation analysis (Fig. 5b). The resulting PC-1 vs. PC-2 scores calculated for the region of the most intensive loadings (at 1030 cm^−1^) give 98% of total variance (PC-1 plus PC-2) of the analyzed data set. This demonstrates excellent separation of the studied bacteria, in one sample test, into three separated clusters corresponding to the *S*. Typhimurium, *L. monocytogenes*, and *C. sakazakii*, respectively (Fig. 5c and ESM Fig. S5) and the ability of the SERS technique combined with PCA to identify the bacteria species according to ISO standards. Moreover, the validation of the PCA method used for identification of food-borne bacteria for five food matrices was performed. In the first step PCA for *Salmonella* Typhimurium, *L. monocytogenes*, and *C. sakazakii* from a selected food medium (five food samples were studied and a total 600 SERS spectra were collected — 40 SERS spectra for each bacterial species) was used to build the PCA model. Then the additional data of the test sample (external food sample with known bacterium identified by ISO method) was introduced into this model. The calculated PCA scores are included in Fig. 5 as asterisks. Three test samples are located in the clusters of the model PC scores corresponding to particular bacterial species. These results highlight the analytical potential of the SERS technique combined with PCA for food-borne bacteria identification.

Additionally, it should be noted that all calculated PC scores are clustered with large distances among particular clusters (*S. *Typhimurium, *L. monocytogenes*, and *C. sakazakii*, e.g., see Fig. 5). At the same time the distances between the calculated scores in each cluster are very short. There are no scores with wrong assignments. Thus, the sensitivity and specificity of the combined SERS and PCA methods are very high (for more information, see ESM).

## Conclusions

The results obtained in the present study demonstrate that SERS is a powerful technique for the detection and identification of pathogenic bacteria in food samples and can be introduced into ISO standards as an alternative method. This strategy enables one to avoid or skip the time-consuming methods routinely used in the laboratory and reduces the time of analysis from 6 to just 2 days. In the presented SERS technique the long, time-consuming incubation required by standard ISO procedures was reduced and the direct SERS analysis of bacteria colonies cultured on agar with selective media was performed. PCA calculations were used to demonstrate the impact of this new approach of the SERS strategy for food-borne bacteria, namely *S. enterica*, *L. monocytogenes*, and *C. sakazakii* identification in selected food matrices (salmon, eggs, powdered infant formula milk, mixed herbs) with 98% of accuracy in only 48 h. The research presented here should open a new path in microbiological diagnostics. It is believed that the proposed SERS-based method can in the future become a robust tool for identification of pathogens in the food industry.

## Electronic supplementary material

Below is the link to the electronic supplementary material.

ESM 1 (PDF 1281 kb)
