# Supplementary material for: Surface-enhanced Raman spectroscopy introduced into the International Standard Organization (ISO) regulations as an alternative method for detection and identification of pathogens in the food industry
Source: Anal Bioanal Chem. 2016 Dec 21;409(6):1555–67. doi: 10.1007/s00216-016-0090-z (PMC5306343; doi:10.1007/s00216-016-0090-z)
Supplement: Supplementary file 1 — (PDF 1281 kb) [file 216_2016_90_MOESM1_ESM.pdf]

Analytical and Bioanalytical Chemistry

Electronic Supplementary Material

Surface-enhanced Raman spectroscopy introduced into the International Standard Organization (ISO) regulations as an alternative method for detection and identification of pathogens in the food industry

Evelin Witkowska, Dorota Korsak, Aneta Kowalska, Monika Księżopolska-Gocalska, Joanna Niedziółka-Jönsson, Ewa Roźniecka, Weronika Michałowicz, Paweł Albrycht, Marta Podrażka, Robert Hołyst, Jacek Waluk, Agnieszka Kamińska

1. Characteristics of *Salmonella* spp., *L. monocytogenes,* and *C. sakazakii*

Salmonella enterica common bacteria found in rotten or unwashed food is one of the most important foodborne pathogens worldwide and the second most frequently reported zoonotic agent in the European Union (EU) after thermotolerant *Campylobacter*. In 2014, a total of 88,238 confirmed salmonellosis cases were reported by 27 member states (MS) of the European Union, resulting in a notification rate of 23.4 cases per 100,000 population [28]. Most people infected with *Salmonella* develop diarrhea, fever, and abdominal cramps. The *Salmonella* infection may spread from the intestines to the blood stream, and then to other body sites and may even cause death [29]. Therefore the fast and simple detection of *Salmonella* in food is needed.

Another serious infection is listeriosis usually caused by eating food contaminated with *L. monocytogenes*. The disease primarily affects newborns, pregnant women, older people, and adults with weakened immune systems. A person with listeriosis usually has invasive infection. Pregnant women may experience fever and other non-specific symptoms, such as fatigue and aches, followed by fetal loss or bacteremia and meningitis in their newborns. Immunocompetent people may experience acute febrile gastroenteritis [31]. In 2014, 27 MS EU (Member State of the European Union) reported 2,161 confirmed human cases of listeriosis in Europe. The EU notification rate was 0.52 cases per 100,000 population which represented a 30 % increase compared with 2013. There was a statistically significant increasing trend of listeriosis over 2008-2014. The highest specific notification rate being observed in Denmark (1.64) and the lowest in Romania (0.03) [28]. Listeriosis represents a serious public health problem since it is fatal with a probability around 20 % of cases during the last two decades [32]. *L. monocytogenes* bacteria are the most important causes of death from foodborne infections in industrialized countries [28]. A total of 210 deaths due to listeriosis were reported in Europe in 2014. This was the highest number of deaths observed

between 2009 and 2014 (annual average: 163). The highest number of fatal cases was reported in France [28].

Cronobacter sakazakii, formerly *Enterobacter sakazakii* (according to an old system of nomenclature), is a germ that can live in very dry places. It has been found in dry foods, like powdered baby formula, powdered milk, herbal teas, herbs, and starches. In babies, *C. sakazakii* germs usually get in the blood or make the lining of the brain and spine swell (meningitis). Up to 4 out of 10 babies with meningitis from *C. sakazakii* can die. Infections caused by *Cronobacter* spp. are also dangerous for older people and people whose bodies have trouble fighting germs, like people with HIV, organ transplants, or cancer [33]. The fast detection of *C. sakazakii*, especially in powdered milk and powdered infant formula, is very important.

2. ISO standards versus SERS-based methodology

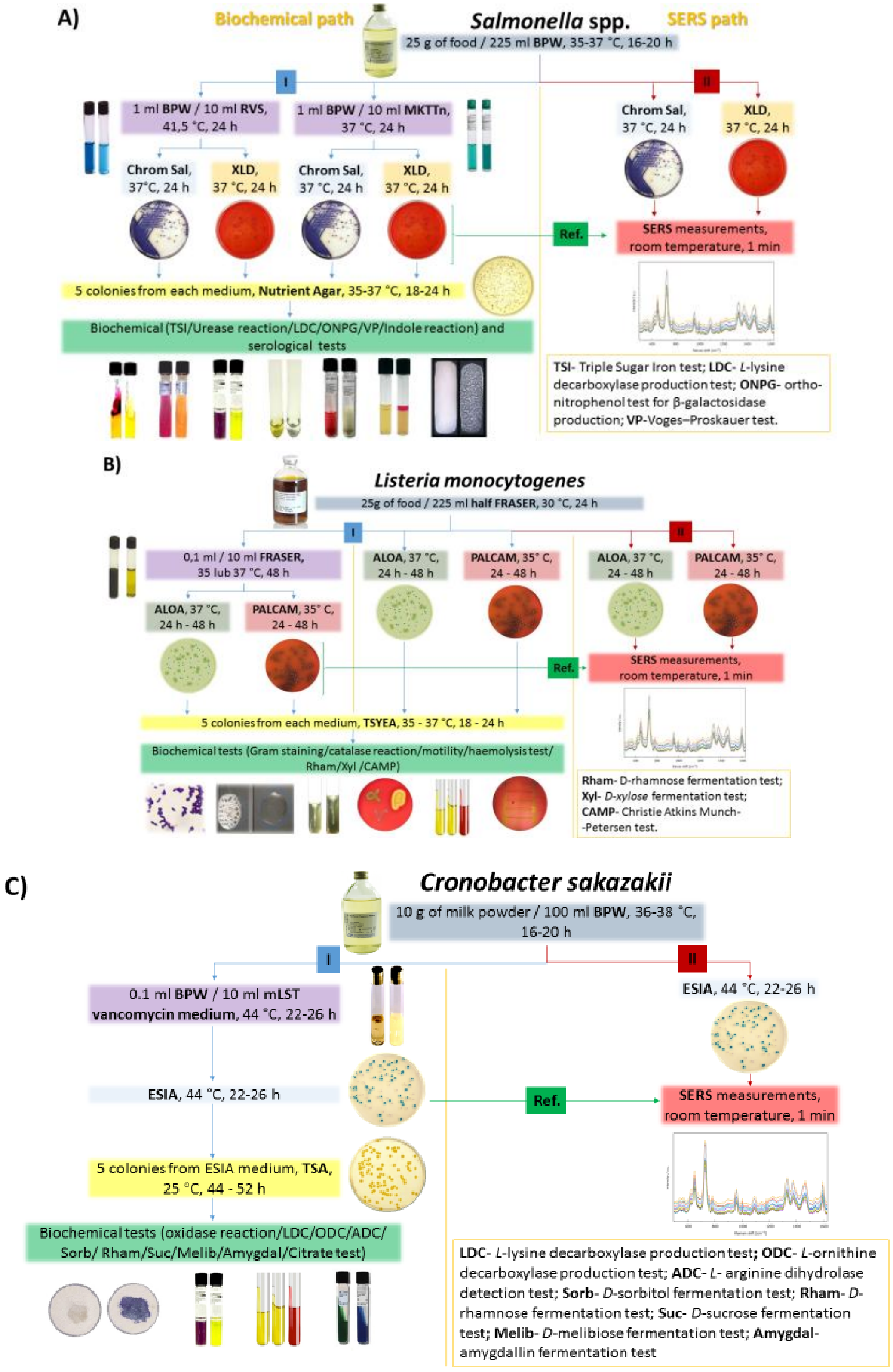

Fig. S1 The detailed schemes representing the different paths applied for (A) *Salmonella* spp., (B) *L. monocytogenes* and (C) *C. sakazakii* detection from food samples

3. Chemometric analysis

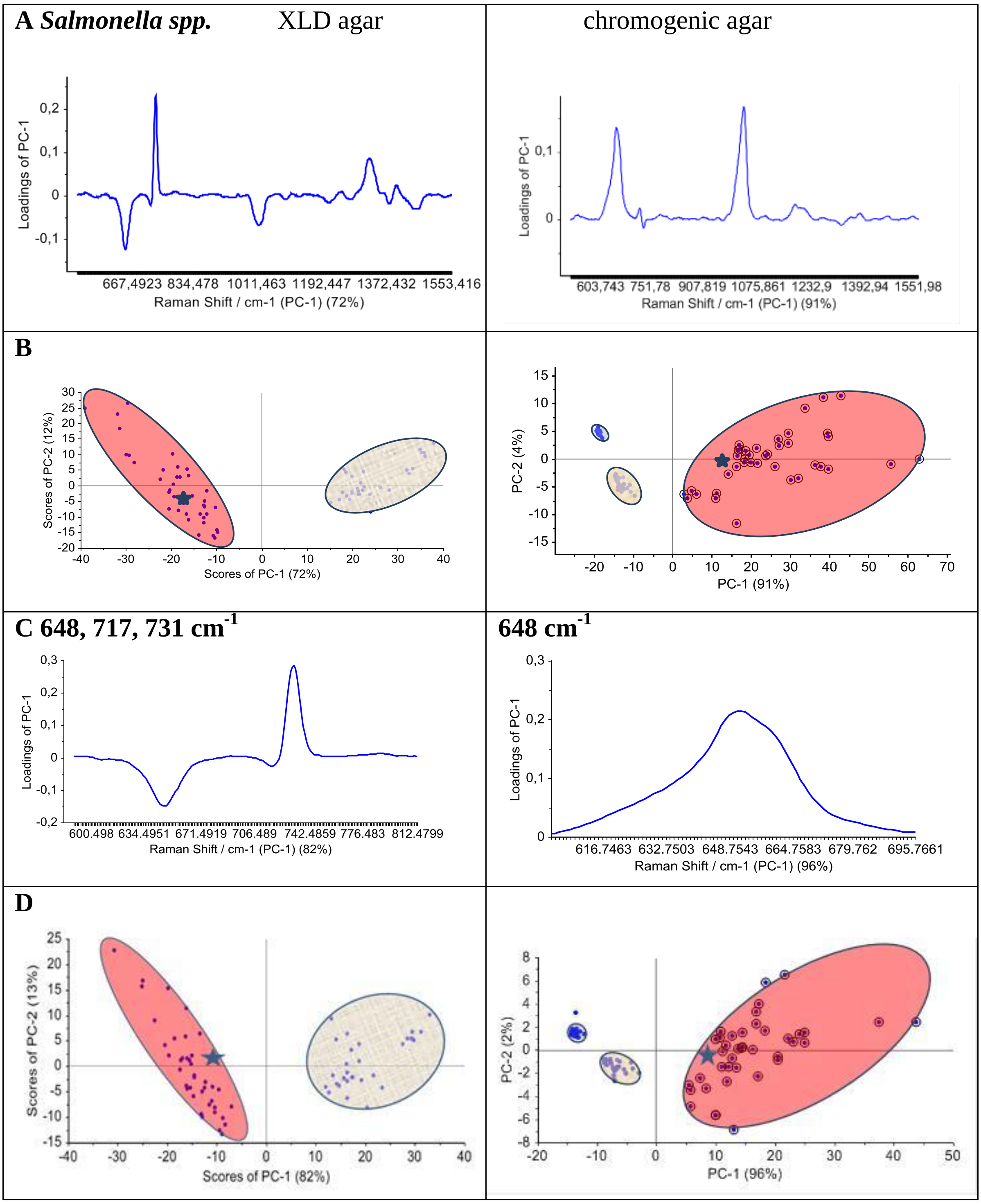

Fig. S2 The calculated PCA analysis for *Salmonella* spp., (A) PC-1 loadings plot in the whole region (650-1600 cm^{-1}) for both XLD and chromogenic agars. (B) PC-1 versus PC-2 scores, both for the same whole region, (C) PC-1 loadings plot, and (D) PC-1 versus PC-2 scores, for the selected regions of 648, 717, 731 cm^{-1}. The asterisks stand for the PC scores calculated for SERS spectra obtained according to reference paths; Fig.1

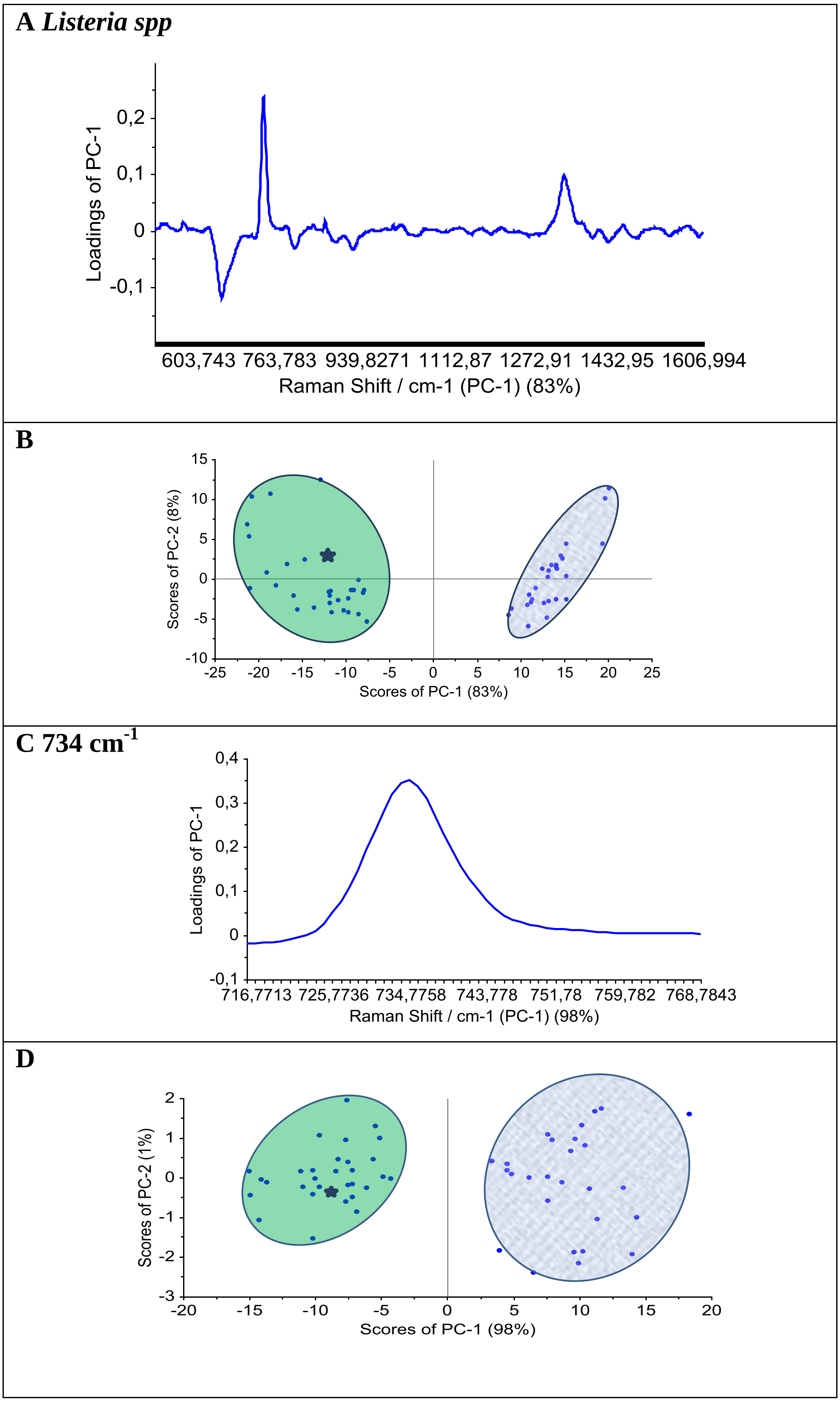

Fig. S3 The calculated PCA analysis for *Listeria* spp.: (A) PC-1 loadings plot and (B) PC-1 versus PC-2 scores, for the whole region, (C) PC-1 loadings plot, and (D) PC-1 versus PC-2 scores, for the regions of 734 cm^{-1} band. The asterisks stand for the PC scores calculated for SERS spectra obtained according to reference paths for test samples; Fig.1

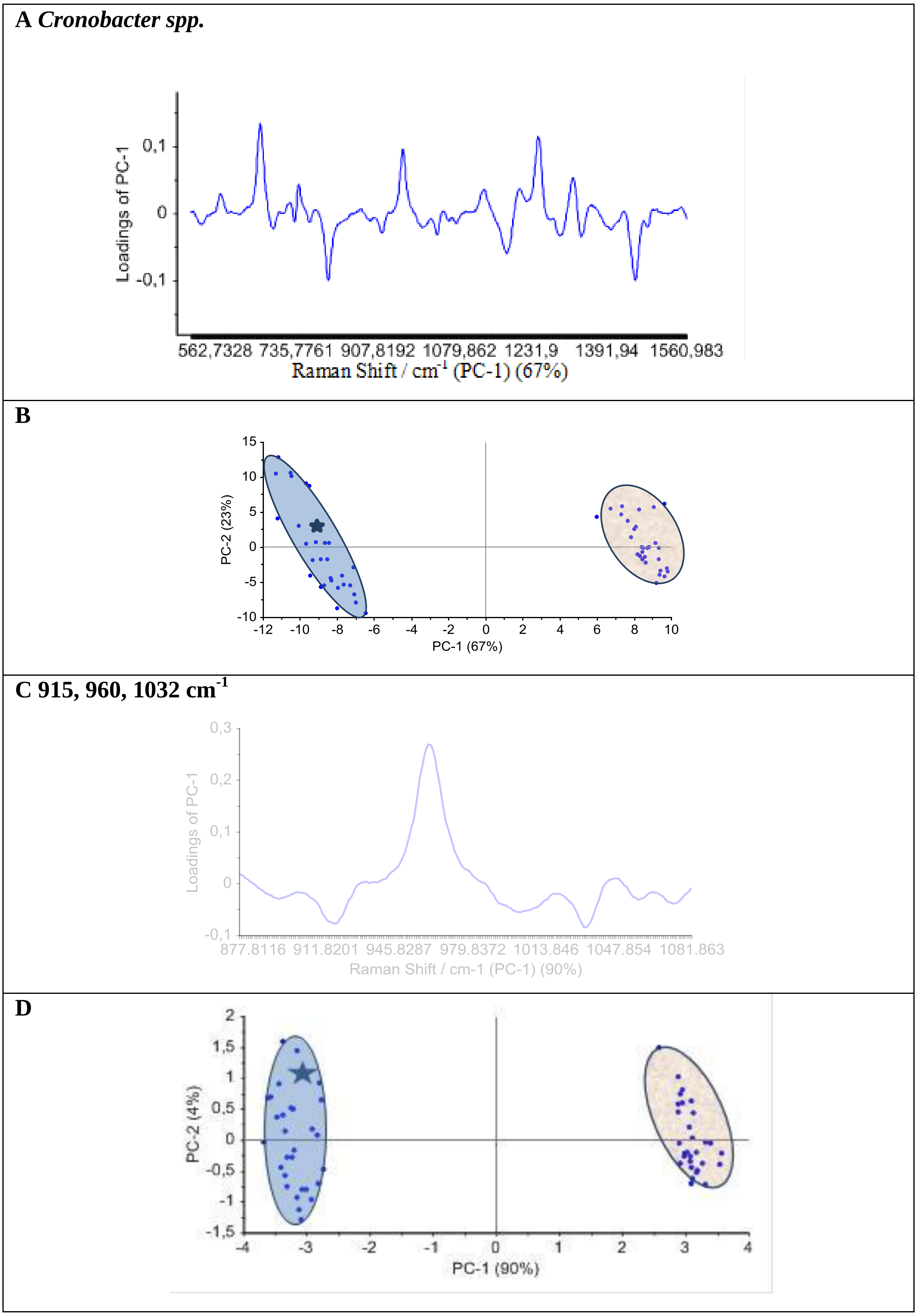

Fig. S4 The calculated PCA analysis for *Cronobacter* spp.: (A) PC-1 loadings plot and (B) PC-1 versus PC-2 scores for the 650-1600 cm^{-1} region, (C) PC-1 loadings plot, and (D) PC-1 versus PC-2 scores, for the regions of bands at 915, 960, 1032 cm^{-1}. The asterisks stand for the PC scores calculated for SERS spectra obtained according to reference paths for test samples; Fig.1

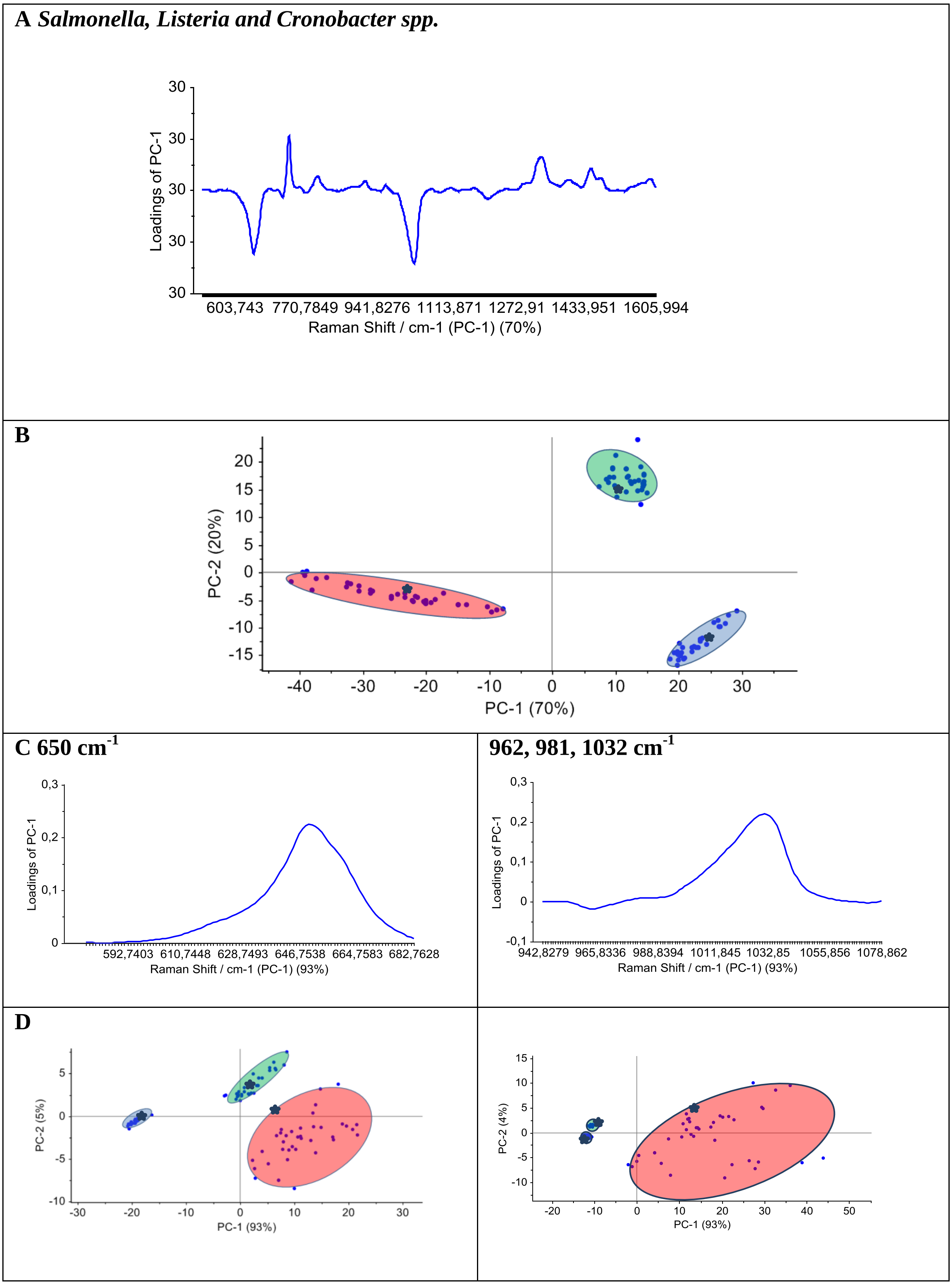

Fig. S5 The comparison of the calculated PCA analysis for three *Salmonella, Listeria and Cronobacter spp.* bacteria.: (A) PC-1 loadings plot and (B) PC-1 versus PC-2 scores for the whole region, and (C) PC-1 loadings plot with (D) PC-1 versus PC-2 scores, for the regions of band at 650 cm^{-1} and bands at 962, 981, 1032 cm^{-1}. The asterisks stand for the PC scores calculated for SERS spectra obtained according to reference paths for test samples; Fig.1

4. Validation of the PCA model

For PCA calculation the Unscrambler@ software were used. In that software we asked for cross-validation of obtained results by uncertainty test (using the optimal number of PC). It is common practice to use cross-validation for determining the number of components and then use that number in further modeling. In our case the calculated scores have been used for building a classification model using principal component regression. The built model performs very well with an R-squared of 0.99 and correlation coefficient of 0.95 (see Fig. S6, Supplementary Materials) for adjusted P-value (conservatively) to 0.05. Our calculations proved that the validated data (cross-validation data) and calibrated (for p-value) are in the excellent agreement (see Fig. S6). Moreover, the predicted and the reference values of PC components are also highly correlated (see Fig. S6). Therefore the proposed model is rational for the classification purposed of our studied data.

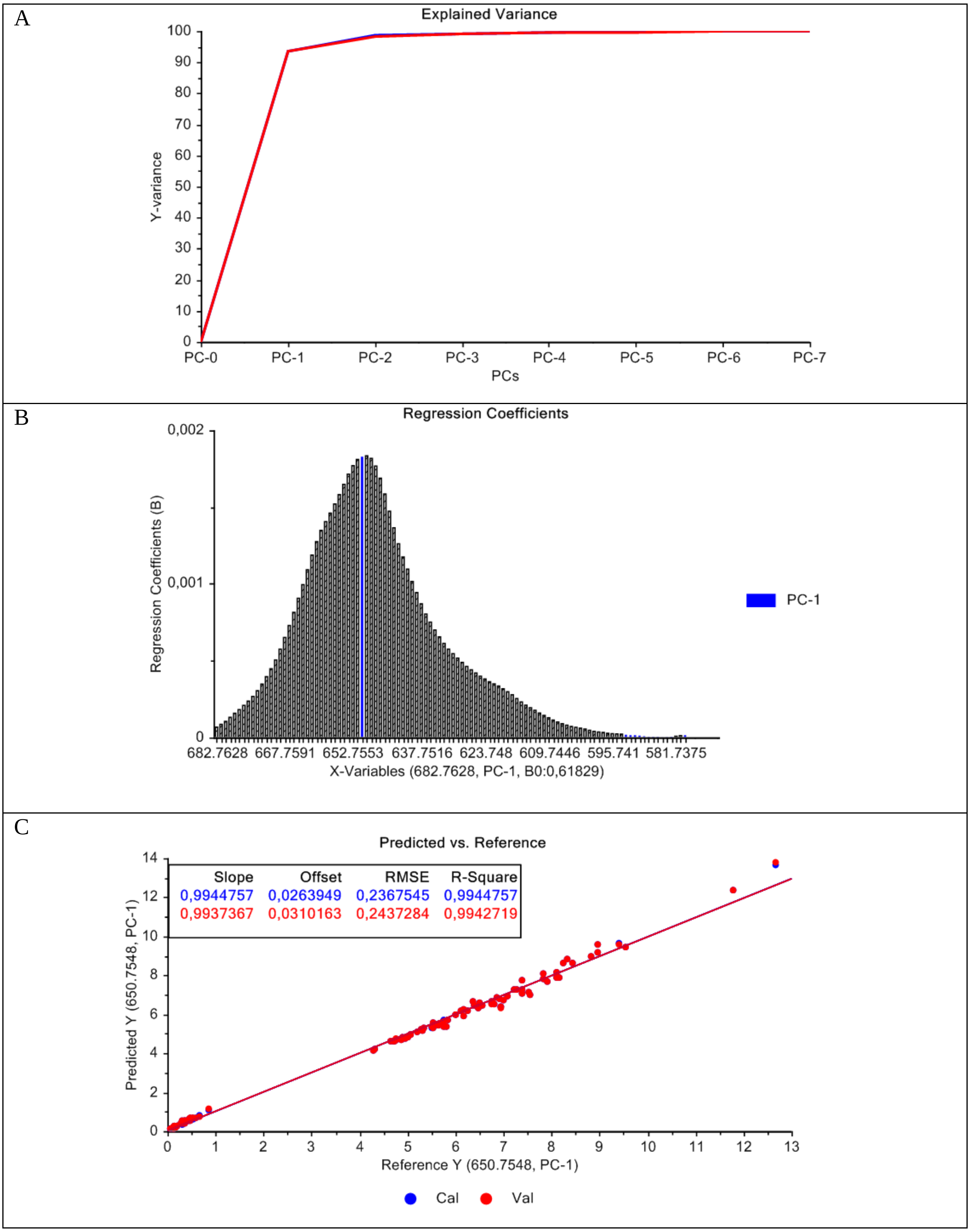

Fig. S6 The figures present given number of PCs used for calculation versus the Y-variance and gives the important number of components (A), the regression coefficient calculated for PC-1 component (B), and The biplot of predicted versus reference PCs values together with RMSE and R-Square (C)

5. **Sensitivity and specificity of used method**

According the PCA calculation all obtained PC scores were nicely clustered with large distance among clusters (*S. Typhimurium, L. monocytogenes and C. sakazakii* , e.g., see Fig. 5). At the same time the distances of the calculated scores in each cluster are very short. There are no scores with wrong assignments. Thus, the sensitivity of used combined methods (SERS and PCA) are very high. It is possible to calculate the sensitivity and specificity according to the formula:

Sensitivity = TP / (TP + FN)

Specificity = TN / (TN + FP)

where TP and TN are the numbers of true positive and true negative results, respectively; FN and FP are the numbers of false negative and false positive results, respectively.

Based on the above equations and the gathered data (with no wrong clustering) the calculated both sensitivity and specificity give 100%. Taking into account that all data in the manuscript are very limited in comparison to the real condition, we are far long from such rigorous statement, thus we decided not to introduce that values to the manuscript.

6. Reproducibility of bacterial SERS signals

The reproducibility of recorded SERS signals plays a crucial role in the analytical and biomedical applications of SERS technique. The average standard deviation (Av. STD) of the SERS signals three bacteria *Salmonella Typhimurium*, *L. monocytogenes* and *C. sakazakii* were calculated and presented in the Table S1.

Table S1 The Av.STD of the selected intensities of SERS signals of *Salmonella* Typhimurium, *L. monocytogenes* and *C. sakazakii* recorded from 30 different spots within the same sample

Bacterial species	Selected bands [cm^{-1}]	Av.STD (%)
S. Typhimurium	1380	20
L. monocytogenes	651	16
C. sakazakii	732	15

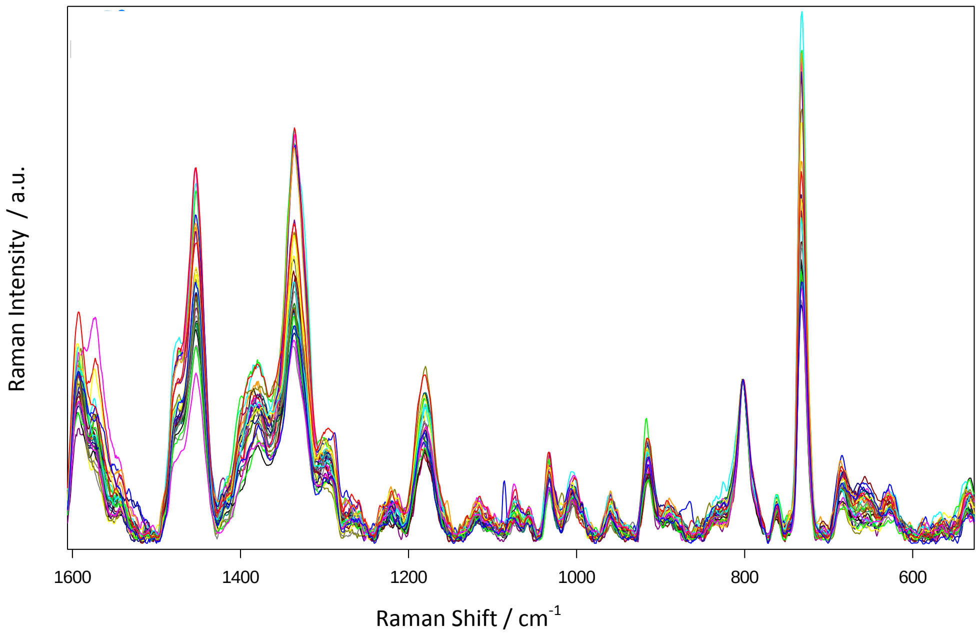

Fig. S7 SERS spectra of *C. sakazakii*, recorded from different spots within the same sample. The excitation wavelength was at 785 nm, laser power was 5 mW, and acquisition time was 60 s
